# “I Feel Like I Work Full-Time for Parkinson’s”: A Longitudinal Interpretative Phenomenological Analysis of the Experiences of Parkinson’s Informal Caregivers during COVID-19 in England

**DOI:** 10.3390/neurosci4020011

**Published:** 2023-05-19

**Authors:** Ian W. Garner, Craig D. Murray, Fiona J. R. Eccles, Nicolò Zarotti, Jane Simpson

**Affiliations:** 1 Division of Health Research, Lancaster University, Lancaster LA1 4YW, UK; 2 Department of Clinical Neuropsychology, Manchester Centre for Clinical Neurosciences, Salford M6 8HD, UK

**Keywords:** COVID-19, interpretative phenomenological analysis, longitudinal, Parkinson’s, caregivers

## Abstract

While the direct effects of COVID-19 caused widespread global suffering and death, the indirect impacts—via public health preventative measures and a reduction in health and social care services—were also devastating for many. More recently, it has also become increasingly apparent that such measures have had disproportionate effects, exacerbating existing health inequalities. For caregivers of individuals with chronic illness, the effects have been marked and particularly so for informal caregivers of individuals with complex neurodegenerative conditions such as Parkinson’s. Nine informal caregivers (all partners: three men and six women) of individuals with Parkinson’s in England took part in individual semi-structured interviews on two occasions (December 2021/January 2022 and May 2022). The interviews focused on their experiences of supporting their partner, their own challenges, and how these evolved and changed post-lockdown. Interpretative phenomenological analysis was used to inform the methodology and analysis. Four themes were identified: (i) lockdown-induced revolution and evolution of the relationship dynamic with partner; (ii) fighting to be seen, heard, and understood in healthcare encounters; (iii) making sense of, and adapting to, risk in a time of COVID-19; and (iv) managing isolation and need for support during and after lockdown. The negative effects from both the lockdowns and the depletion of usual health and support services were apparent for all participants. Existing vulnerabilities from being a carer of an individual with complex needs were also exacerbated. As caregivers worked hard to mitigate these effects for their partners as much as possible, the long-term impact of such intense psychological and practical effort was often considerable. Ultimately, a simple restoration of pre-lockdown support levels may be insufficient to facilitate a successful return to optimal levels of support and wellbeing.

## 1. Introduction

When COVID-19 emerged, countries across the world initiated ‘lockdowns’ to protect individuals and prevent the spread of the virus. In England, the following lockdowns were introduced [1]: March 2020–August 2020 (Lockdown 1); November 2020–December 2020 (Lockdown 2); January 2021–June 2021 (Lockdown 3). Mandatory measures during these lockdowns included the closure of all schools and non-essential shops, social distancing restrictions, and a requirement to wear face masks when entering closed environments. Additionally, individuals who were categorised as ‘vulnerable’ were instructed to stay at home and depend on others for their daily necessities. Among the individuals classified as vulnerable were those with Parkinson’s disease (henceforth referred to as ‘Parkinson’s’)—a chronic neurodegenerative condition which initially affects motor control but which can also be associated with other cognitive and psychological difficulties.

An estimated 121,000 individuals are currently living with Parkinson’s in England [2]. Of these, approximately 90% receive varying levels of support primarily from their partner (referred to as ‘informal caregivers’—for the sake of conciseness, the term ‘caregivers’ will be used to refer to informal caregivers from this point on in the present article) for everyday tasks such as shopping, cooking, and maintaining personal hygiene [3]. Prior to the COVID-19 lockdown measures, caregivers were estimated to provide an average of 50 h a week of care for their partners [3], which significantly increased during the pandemic and negatively affected their quality of life and wellbeing [4]. In fact, a rapid review of 16 studies by Lorenz-Dant and Comas-Herrera [5] found that, within just eight months of the first lockdown being implemented across the world, caregivers of adults with long-term needs (such as mental and physical impairments) reported increased feelings of loneliness, burden, and stress, as well as sleep deprivation, irritability, and anguish. Similarly, a scoping review by Brooks and colleagues [6] found that, during the first worldwide lockdown period, caregivers of people with Parkinson’s (PwP) were faced not only with a worsening financial situation but also increased worry and uncertainty, feelings of guilt, grief, and frustration, and negative changes in lifestyle. In addition, a survey of 533 UK-based PwP and 189 caregivers in summer 2021 (after all UK lockdowns had ended) suggested that the mental wellbeing of the latter was even poorer than that of the PwP [7].

While a number of quantitative studies have been carried out on the challenges faced by PwP and their caregivers during the COVID-19 pandemic (e.g., [8,9,10]), qualitative research on this topic appears to be much sparser [6]. To our knowledge, only one study to date [11] has considered PwP’s caregivers’ perspectives on their experiences related to COVID-19 in the UK, finding that individual vulnerabilities to COVID-19, engagement with healthcare services, and home maintenance and activities of daily living all represented major themes. However, this study collected data from a single time point in April 2021 and therefore was not able to elaborate on how these lived experiences evolved following the end of the lockdowns in the UK. 

Consequently, the current study employed a longitudinal, qualitative methodology to explore caregivers’ lived experiences of supporting both their partners and their Parkinson’s during lockdown, the effects of these experiences on caregivers, and how these evolved after the lockdowns ended.

## 2. Materials and Methods

### 2.1. Design

An interpretative phenomenological analysis design was adopted [12]. Data were collected through individual semi-structured telephone interviews on two separate occasions—first between December 2021 and January 2022 and again during May 2022.

### 2.2. Sampling and Participants

Participants were recruited through the research network of Parkinson’s UK (the country’s largest charity supporting people with Parkinson’s and their caregivers) as well as a participation database at the researchers’ host institution. To ensure optimal levels of homogeneity and relevance to the study aims [12], participants were required to have provided informal (unpaid) care for their partner with Parkinson’s for a minimum of 12 months prior to lockdown. This allowed participants to consider how lockdown had impacted their experience of supporting their partner’s Parkinson’s.

Ten individuals initially showed an interest in taking part in the study. Of these, nine (three men and six women) eventually agreed to participate. These were all white British and lived in England. Recruitment and the first round of interviews were completed within one month of contacting each participant. See Table 1 for the participants’ demographic information.

### 2.3. Data Collection

Participants were interviewed at two time points. Time 1 (T1) interviews were completed between December 2021 and January 2022. The interview process began with gathering contextual information about the participant and their partner (e.g., age, gender, years since Parkinson’s diagnosis). Following this, interviews focused on understanding participants’ lived experiences of supporting their partner and their Parkinson’s during the lockdowns. Questions aimed to understand how participants’ experiences evolved across each lockdown, with questions initially focusing on perspectives during the first lockdown (March 2020–August 2020) before moving on to the second lockdown (October 2020–December 2020) and, finally, the third lockdown (January 2021–June 2021).

Time 2 (T2) interviews were completed in May 2022 and focused on understanding how participants’ experiences had evolved after all the lockdowns had ended. Parts of the T2 interviews were tailored to each participant based on their experiences discussed at T1.

Semi-structured interview schedules were created for both time points to provide a broad framework for the interview process. At T1, the framework was based on findings by Simpson et al. [13] and covered topics such as experiences of providing care, accessing support, and accessing healthcare during lockdown, as well as the impact of COVID-19 on participants’ health and wellbeing and other factors participants felt were important to note. The T2 interview framework expanded on the previous framework with additional, tailored questions based on topics discussed at T1. All interviews were completed via telephone, audio recorded, and transcribed verbatim. The mean length of T1 interviews was 54 min, while T2 interviews lasted 36 min on average.

### 2.4. Data Analysis

Interpretative phenomenological analysis (IPA) was adopted in the present study. The philosophical underpinnings of IPA are rooted in phenomenology (i.e., understanding lived experiences) and hermeneutics (i.e., interpreting participants’ sense making of their experiences [14]). Moreover, IPA has an idiographic focus that explores similarities and differences in the experiences of a small (typically 3–10), specific, and homogenous sample [12].

IPA often involves data collection from one-off, individual interviews. However, it is also suitable for longitudinal analyses [15]. To conduct longitudinal IPA, the researcher is required to capture the temporal experience as it evolves over time [16]. IPA’s focus on understanding the sense making of participants makes it an ideal approach to explore how temporal experiences vary [17]. In the current study, this is represented by caregivers’ experience of supporting their partner with their Parkinson’s during and after lockdown.

T1 data analysis followed Murray and Wilde’s [18] guidelines for IPA. First, each transcript was reviewed several times to aid familiarisation with the data and analysed in isolation without considering themes derived from other interviews. Transcript notations were made on information relevant to the research aim and included direct quotations (e.g., ‘I feel I work full-time for Parkinson’s’), paraphrases of participants’ descriptions (e.g., ‘participant fighting to access healthcare’), and interpretations of participants’ sense making (e.g., ‘working for Parkinson’s, not supporting husband?’). Each notation was then grouped based on the subject discussed by participants. This process was repeated across all transcripts. Grouped notations were then merged on a cross-participant basis, and themes were derived based on the similarities and differences between participants’ experiences on specific topics. Finally, an interpretative narrative summary was developed for each cross-participant theme.

Analysis of T2 data followed the ‘themes spanning time’ approach described by Farr and Nizza [16], whereby T2 data analysis focused on understanding how T1 themes had evolved at T2 as opposed to discovering new themes. Finally, analyses across both time points were synthesised into single longitudinal themes which showed the evolution of participants’ sense making of their experiences and how and why the trajectory of experiences varied between participants.

### 2.5. Ethics

Prior to beginning the data collection, this study was approved by the host university’s Research Ethics Committee.

## 3. Results

Four overarching themes were identified: (1) lockdown-induced revolution and evolution of relationship dynamic with partner; (2) fighting to be seen, heard, and understood in healthcare encounters; (3) making sense of, and adapting to, risk in a time of COVID-19; and (4) managing isolation and need for support during and after lockdown. Each theme is outlined below, along with data excerpts from both data collection points (T1 and T2).

### 3.1. Theme 1: Lockdown-Induced Revolution and Evolution of Relationship Dynamic with Partner

In this theme, we explored how the lockdowns changed and developed the relationship between the person with Parkinson’s and their partner. Although changes in the spousal relationship varied between participants, for all, their relationship dynamic did not remain the same. A number of factors were evident which influenced the extent and type of change—including the availability of healthcare services, pre-existing tendencies, the severity of their partners’ Parkinson’s, and their partner’s own coping strategies.

In terms of reasons for change, for some, this was the result of diminished input from a range of healthcare professionals, which led partners to assume the daunting responsibility of a complex, multiskilled, full-time carer role:

*And I think it was the responsibility that I had [caring for her husband during lockdown] that really frightened me because I thought it’s up to me now to keep [name] well and exercise so I became his speech therapist, his physio, his OT, his pharmacist, everything*.

(Gemma (All names have been changed to preserve anonymity), T1)

Moreover, Gemma’s description of herself as becoming her husband’s ‘everything’, yet indicating only a range of medical roles, is reflective of the change in the needs of her husband during the lockdowns from that of needing a partner to that of needing a skilled carer. The relationship shifts towards becoming more of a carer during lockdowns appeared to be due to caregivers’ prior reliance on healthcare professionals in supporting their partner. For instance, some felt more like a carer during lockdown despite minimal lockdown-induced changes:

*We just, we carried on, we do, you know, our meals, we have… I tend to get them organised, yeah, I mean, I do think that I’m more of a carer now than I ever was, but it’s not to say he couldn’t do it*.

(Alice, T1)

Following the end of the lockdowns, participants expressed an evolution of their supportive role, with their role of full-time carer switching from being partner-centred to one in which Parkinson’s dominated their attention. As one noted:

*I feel like I work full-time for Parkinson’s, and so I don’t have time, very little time for myself, other than when [name’s] gone to bed, and so I probably stay up later than I should, reading or knitting or something*.

(Gemma, T2)

Lockdown appeared to catalyse the deleterious progression of Parkinson’s in caregivers’ partners but also heralded PwP’s mental shift towards a less active approach to managing the condition. Some caregivers attributed this change to inactivity during lockdown:

*And it [lockdown] has speeded along [Partner]’s deterioration because he doesn’t have to make an effort to go out any more, he doesn’t have to speak with people. So now he would prefer not to bother*.

(Janine, T2)

Accelerated Parkinson’s progression meant that the participants could not do the same regular activities with their partners as they could pre-lockdown, and this almost felt as if Parkinson’s had created an insurmountable barrier between them:

*I’m watching him slip away through my fingers, really, and trying to advise him, trying to motivate him*.

(Janine, T2)

For some participants, Parkinson’s becoming more dominant represented a shift in their perception of their caring role, one in which closeness was transformed, discussed, and managed together:

*Yeah, in some ways, it’s almost like being a parent [caring for husband], and that’s what is really difficult, because obviously, you know, we started out as an equal partnership, and now it’s, like, it’s a bit like being on a seesaw really, you know, I’m either at the bottom or the top. And we can never be balanced, because I’m making the decisions and saying, “Well, we can do this, we can’t do that”. Yeah, it is difficult*.

(Rachel, T2)

In contrast, others spoke about how their relationship with their partner deepened because of lockdown:

*I think we’d just got into a routine. I think we’d just got into a routine of being just us, and just walking, and having our dinner, and just doing things, just the two of us. And I think we just got into a routine of doing that. I think there wasn’t any pressure on us to be here or be there*.

(Patrick, T1)

When all lockdowns ended, participants were eager to retain the togetherness developed with their partners in the widening of horizons that the easing of restrictions afforded:

*We still tend to do most things together, you know, for instance, we’re planning to go up to X in the [Location], one of our favourite places, and do, this weekend, something like a 15-mile walk*.

(Paul, T2)

However, participants also recognised that their togetherness was predicated on their partners retaining their capacity for independent activity and minimising the effects of Parkinson’s:

*So, I don’t really want to feel like I have… I want him to be independent and, yeah, but the other day, he did actually freeze, and I thought, “Oh I’d better help him with his coat. I’ll have to help him”. So yeah. I still don’t want to, I don’t see myself as that, but it didn’t feel nice. And I was… I suppose I did turn into that carer the other day and it felt horrible*.

(Rachel, T2)

Overall, lockdown restrictions appeared to affect participants differently—either by creating a divide between the participant and their partner or bringing them closer together. Furthermore, for many, the end of lockdown restrictions did not allow participants to revert to their pre-lockdown relationship dynamic, suggesting that lockdown had a lasting and potentially permanent effect on the quality and type of relationship between participants and their partners.

### 3.2. Theme 2: Fighting to Be Seen, Heard, and Understood in Healthcare Encounters

Caregivers recounted their experiences of liaising with healthcare professionals during and after lockdown and how lockdown-induced changes shifted the priorities of healthcare professionals, leaving participants feeling as if their struggles in supporting their partners during lockdown were not understood.

When lockdown restrictions were implemented in the UK, the focus of healthcare professionals was perceived to shift towards COVID-19, and participants felt that the challenges of being a carer for PwP were not fully appreciated:

*Yeah, well, as a carer you’re invisible a lot of the time. [we had contact from the surgery] to say we could book his flu [jab]. So, I phoned them and I booked it and I said I will have to bring [name] and I’m his wife, I’m his full-time carer, but I haven’t had a letter yet which, we’re the same age, I haven’t had a letter yet and they said “we can’t book yours until you get your letter”. And it just makes you feel… sometimes it’s such an effort. I feel as though you have to pick your fights, but I do feel very strongly about that because if I get the flu, we won’t manage*.

(Gemma, T1)

Gemma’s description of herself as invisible in the eyes of healthcare professionals, along with the need to ‘make’ her local GP practice staff understand her ‘fight’, conveys her perception that healthcare professionals failed to consider her strain in supporting her partner. One reason provided for these sorts of difficulties was the lack of opportunity to express their concerns with the healthcare team who would normally support them:

*That was the frustrating part of COVID. I mean, not being able to talk about [Partner’s] condition in a, you know, in a room with another healthcare professional who knows him, you know, or actually can see those subtle changes, you know… I would say since he’s been going back to the hospital, you know, they’ve changed his medication and they’ve increased his medication, and he needed it, you know. And as far as I’m concerned, you know, that was frustrating*.

(Patrick, T1)

The intertwinement of caregivers and partners in managing Parkinson’s appeared to remain after lockdown ended. Having experienced lockdown together with their partners, participants felt more connected with Parkinson’s, as if it were as much a part of their daily lives as it was their partners’. For example, one caregiver spoke of her unity with her partner over Parkinson’s decision making and emphasised that she lived with Parkinson’s together with her husband but that this was not recognised by healthcare professionals:

*I do get very frustrated and I am very irritable sometimes, but when we have so many, you know, seeing lots of different professionals at different times, and I feel…and they will always start off and want to talk to [name], but it’s so exhausting for him that we have an agreement, and I’ll say to [name], “Would you like me to explain how you’ve been, [name], is that okay?” and then I just bring him in all the time, rather than me just talking over [name]. But it’s his…well, it’s our life*.

(Gemma, T2)

Healthcare professionals were perceived as viewing caregivers’ input as relatively unimportant during consultations. This was considered by caregivers as negatively affecting their partner’s care as only a more limited amount of relevant information was being solicited. Caregivers felt they could provide novel, valuable knowledge and expertise on their partner’s condition:

*I think they [consultants] could have done a little bit more for him. They just take it, because he seems to be a strong person, that he doesn’t need it as much, and I’m sorry, they don’t know him, you know, they’ve not looked at the situation properly and taken into account what he was going through with me*.

(Catherine, T2)

As a result, caregivers spoke of their frustration with healthcare services and the impact on their wellbeing, the effects of which were highlighted by participants seeking counselling to cope with their frustrations:

*I did, actually, realise that I need to talk it [anxiety of husband not receiving suitable Parkinson’s support] through with someone and I’ve done that. I requested some counselling and had to have it privately because I needed to offload*.

(Janine, T2)

Participants felt that healthcare professionals failed to understand how they, as caregivers, lived with Parkinson’s as much as their partners. They felt as though healthcare professionals disregarded their difficulties in living with their partners’ condition through and beyond the lockdowns, which, ultimately, had negative effects for both the PwP and the caregiver.

### 3.3. Theme 3: Making Sense of, and Adapting to, Risk in a Time of COVID-19

The third theme focuses on how the restrictions and official guidance that comprised the UK government’s early response to COVID-19 presented caregivers and their partners with novel dilemmas on how to behave and protect themselves and hence make sense of and adapt to perceived risks. Participants interpreted the changing guidance differently, with some following the guidance at all stages, others being more ready to follow the more (as opposed to less) stringent guidance, and others calculating personalised risk assessments.

Some participants were very dependent on official COVID-19 guidelines to make informed decisions. Moreover, as they felt their partners were vulnerable to adverse effects if they contracted COVID-19, they chose to remain under strict guidelines:

*I mean, it’s, like, sometimes my partner will say to me, “Shall we just go out and do such and such,” just for a run in the car and what have you, and sometimes I don’t want to do it, I don’t want to go out of the house. And it’s not because of going near anybody. I don’t really know sometimes why. I suppose I feel secure in the house, I feel comfortable and safe in the house*.

(Sandra, T1)

For these caregivers, lockdown was a period when they had little control over their daily lives and lived with a constant fear of their partner contracting COVID-19 and/or contracting it themselves. Moreover, official guidance was their only viable pathway for navigating lockdown, and to diverge from the guidance was perceived as diverging from safety. When the lockdowns ended, some participants remained reliant on official guidelines, although these were then about reintegrating into society:

*So, I think after that [lockdown] mind, it’s been a bit of shoulder shrugging, like, “Well, so what? We’re going out now”. And like I don’t wear a mask anymore*.

(Sandra, T2)

For Sandra, her belief that her home was her only safe place during lockdown was predicated on her fearing contracting COVID-19. However, when she and her husband contracted COVID-19 and only had ‘mild symptoms’, her fear was lifted. Therefore, she changed her perception, and reintegrating into society became a lower risk; she then fully embraced the freedoms post-lockdown regulations allowed.

In contrast, another participant spoke about her anxiety in relation to leaving lockdown, with her and her husband still wearing masks inside stores and hospitals as they were conscious of contracting COVID-19:

*I’ve had quite a few family members and friends that sort of, you know, have had it, and they’re very good because they will sort of let me know, even our next-door neighbour, she and her little boy had it and she texted me and said, “Just keep away from us because we’ve got COVID and we don’t want to give it to you,” you know. So, I’ve had some good friends that have been protective as well, if they’ve had a cold or whatever, they’ve sort of, you know, said, “Look, I’m not going to come,” or, you know, they’ll phone, that kind of thing*.

(Catherine, T2)

Unlike Sandra, Catherine—who had not contracted COVID-19—felt surrounded by COVID-19, with friends, family, and her immediate neighbours contracting it. Furthermore, their warnings of not wanting to give her COVID-19 and Catherine’s belief that they were being protective of her likely increased her and her husband’s perceived risk with regards to COVID-19, which, in turn, made her more cautious. In contrast, some participants drew on their professional backgrounds and utilised this knowledge in tandem with the generic government guidelines to perform personalised risk assessments for them and their partner:

*Well, one of my postgraduates is epidemiology so I was able to take a reasonable… the actual risk of contracting COVID-19 first time round was actually, was quite infinitely small if you just changed your lifestyle slightly*.

(Paul, T2)

After lockdown restrictions eased, caregivers were concerned about the increased risk of contracting COVID-19. However, unlike during lockdown, their analytical approach to protecting themselves from COVID-19—using risk analyses—wavered due to the emotional toll of lockdown, leading them to perform higher-risk activities:

*We’re on a coach for this tour, so there’s going to be hours and hours on a coach and I don’t think I could cope with wearing a mask all that time. I think I’ll start to feel claustrophobic. They’re not insisting that you do but then I think should we maybe do it just for that protection, you know, so you’re always that little bit nervous about things, you know*.

(Rachel, T2)

For these participants, lockdown was a period during which they had been able to make rational decisions to maximise their safety and minimise the risk of contracting COVID-19. However, the emotional impact of going through lockdown meant that, when restrictions ended, they were more willing to engage with the risk of contracting COVID-19 to improve their wellbeing. Overall, although caregivers’ wellbeing suffered through lockdown because of strict restrictions, having access to professional knowledge in assessing risk during lockdown appeared to provide some with greater freedom to retain control over their daily lives.

### 3.4. Theme 4: Managing Isolation and Need for Support during and after Lockdown

The last theme is concerned with how lockdown restrictions appeared to have had a dual effect on the support networks of a number of caregivers. For some, restrictions reduced access to general social, as well as Parkinson-specific, support, even though some level of social networking was maintained by rekindling and/or building new friendships. This reduction represented the loss of an essential part of their partner’s healthcare support:

*Oh well, we haven’t got that [Parkinson’s nurse access] now [during lockdown] and I know you can ring them, you can call them, but it is… yeah, it was just like a little bit of the jigsaw was missing, and you didn’t quite have everything that you needed*.

(Sandra, T1)

In contrast, those who were fortunate enough to retain access to Parkinson-specific support spoke of the benefits it provided in relation to their wellbeing:

*We were reassured because she was having regular conversations with the Parkinson’s nurse who is first class and my wife, we’ve got other friends have got, you know, who’ve gone through Parkinson’s are friends of ours*.

(Paul, T1)

Parkinson’s support networks comforted participants and gave them the opportunity to discuss issues related to the condition with people who understood their difficulties:

*I think as long as you’ve got the contact with your Parkinson’s nurses and doctors, you know, and they have been very good, they’re on call any time, you know*.

(Rachel, T1)

For most caregivers, their Parkinson’s network represented security—a resource they could utilise if needed. Even when they did not need to contact professionals, knowing it was present was enough to provide comfort. It provided the opportunity to have one-to-one discussions about their concerns with someone who understood them and reduced their feelings of loneliness. Indeed, when lockdown restrictions were implemented, the caregivers who lost this support highlighted how isolated they began to feel, since the only people they could speak to did not fully understand their circumstances:

*I know and people tell me that and say “oh, you know, could you go to bed early or could you get some exercise?” and I say, “well, I’d love to go to bed early but I’ve got to make sure [name]’s in bed first because I can’t leave [name] to switch lights off and make sure the doors are locked or that there’s nothing left on.” And then doing exercise I can’t leave [name] on his own in the house unless I know that he’s completely safe, you know, if he’s asleep. So, if he goes to bed, I’ll whizz out for a quick walk*.

(Gemma, T1)

As lockdowns ended and some caregivers were able to rebuild their Parkinson’s networks, the importance of this type of support was further emphasised:

*Yeah, that’s [meeting neighbours to talk during lockdown] changed now, because you know in the lockdown, people…we meet [different group] on Thursdays, because that’s when the physio is, and while our partners are leaping around, about half a dozen of us sit around chatting about how Parkinson’s is affecting us*.

(Paul, T2)

This appeared to be particularly evident when caregivers were given the opportunity to socialise with other people caring for PwP who could empathise with their difficulties:

*I do have a few friends whose other halves have got Parkinson’s, and so we support each other and we have a moan together, or we text each other, and that’s…that’s really helpful because you know that that other person is going through the same or similar, and so it’s not a case of just moaning, it’s just someone understanding what you’re saying*.

(Gemma, T2)

In contrast to some participants who were especially dependent on healthcare professionals for Parkinson’s support, the end of lockdown restrictions was still met with a reduced availability of support compared to the pre-lockdown period:

*We say we [participant and husband] could paper the spare room with the cancellation letters for outpatient appointments because they make them then they cancel them*.

(Alice, T2)

Similarly, Catherine spoke about her difficulty trying to speak to their consultant about her husband’s medication changes, describing how she and her husband needed ‘a bit of reassurance’ that his medication changes would not adversely affect his health:

*And he’d [husband] sent this information to the consultant’s secretary for her to give it to him for him to look at, and that’s one of the things that he was supposed to be ringing us back on, and of course, he’d also asked a few questions about the new medication they wanted him to go on, and it had just gone on for so long, and I think, you know, that [difficulty contacting the consultant] had a bad effect really in that respect*.

(Catherine, T1)

For these participants, the inability to regain their Parkinson’s support following the end of lockdown restrictions made them feel as though they were still isolating from others due to the impact this had on their mood and wellbeing:

*Okay, I suppose the balance of life with no social life and no recreation, we’re one to one in isolation, and his [husband] mood has slipped quite a lot. So, I don’t feel sometimes that he’s there anymore*.

(Janine, T2)

Ultimately, Parkinson-specific support seemed to play a pivotal role for caregivers of PwP, and its reduced availability experienced by some following the end of restrictions appeared to make them feel as if they were unable to exit lockdown fully.

## 4. Discussion

### 4.1. Summary of Main Findings

In this study, we aimed to understand the lived experiences of caregivers of PwP in England during and after the COVID-19 lockdowns. Four overarching themes were identified: (i) lockdown-induced revolution and evolution of the relationship dynamic with partner; (ii) fighting to be seen, heard, and understood in healthcare encounters; (iii) making sense of, and adapting to, risk in a time of COVID-19; and (iv) managing isolation and need for support during and after lockdown. While some of the pressures and challenges of being a Parkinson’s carer during lockdown in England have also been identified elsewhere [11], the longitudinal aspect of this study allowed greater insight into the evolution of these challenges.

During lockdown, a shift in caregivers’ relationship dynamics with their partner was notable across participants, with some feeling as if the lockdowns had brought them closer together, whereas others felt they had become more of a carer and less a partner. This was consistent with Günther-Bel et al. [19], who also found that marital relationships could either deteriorate or strengthen during lockdown based on their capacity to cope collectively. The direction of the relationship dynamic change was substantially impacted by the severity of participants’ partners’ condition, with a strengthening of the relationship observed in those whose partners lived with earlier-stage Parkinson’s, which may suggest that couples affected by more severe Parkinson’s symptoms were not able to cope collectively but had to adapt individually [19].

This distinction in the role for caregivers appeared to be reinforced after lockdown. In particular, those who noted that the relationship with their partner had strengthened were keen to retain their renewed sense of togetherness, perhaps by remaining in lockdown. On the other hand, the participants who had begun to feel more of a carer than a partner during lockdown felt that this continued after restrictions were lifted, as though their roles had become more formal and focused on Parkinson’s (as a more abstract, less personal disease entity) rather than their partner. For these participants, there was a belief that lockdown had caused damage to their relationship dynamic which might not be reversible.

Theme 2 explored how, during lockdown, many participants felt they experienced difficulties in accessing healthcare services for their partner—and those who did manage to access them found that a strenuous effort was required to obtain appointments. This also appeared to extend to participants themselves, as they experienced their own difficulties in accessing healthcare while caring for their partner. This struggle seemingly added to their feelings of being ‘invisible’, which is consistent with other findings suggesting how caregivers can feel under-recognised, frustrated, fatigued, and despondent [20,21].

Theme 3 outlined how a major part of the role of caregivers was to assess risk during and after the lockdowns and how this ability tended to vary between participants. Indeed, some were able to draw on professional knowledge (via education and/or previous work experience) to determine what daily activities they could do with minimal risk of contracting COVID-19, which gave them a greater sense of control over their daily routine. In contrast, the caregivers who felt they could not assess risk accurately preferred to remain under strict lockdown conditions and avoided public places, which, in turn, made them feel increased anxiety and worsened their wellbeing. These findings are again consistent with previous studies, particularly in light of the well-established positive effects of perceived control on psychological adjustment to and caregiving for neurodegenerative conditions [22,23,24,25,26,27,28] and Parkinson’s specifically [29,30,31,32].

Finally, Theme 4 outlined how lockdown restrictions reduced participants’ access to their Parkinson’s support networks, which, in turn, increased their feelings of isolation (see also [7,33]). The importance of a Parkinson’s support network was also highlighted post-lockdown, with some caregivers emphasising the benefits of being able to discuss their issues with individuals who could empathise with their struggle [25] and, particularly, fellow caregivers [34]. This appeared to be especially important when considering the potential impact of stigma on the psychological wellbeing of PwP [35]. Conversely, the participants who did not manage to regain access to their Parkinson’s support network following the easing of the restrictions described their situation as though they were still in lockdown. While feelings of isolation and need for support are not unusual when caring for individuals with long-term conditions [36], the severe and unique experience of COVID-19 lockdowns and the specific challenges these brought likely increased the need for this sort of support.

### 4.2. Theoretical and Clinical Implications

From a theoretical point of view, the results from this study suggest that support and guidance from official government sources and healthcare professionals should aim to foster a sense of control for caregivers, with a clear recognition of their contribution and importance not only in a time of such unprecedented turmoil, but also in the everyday life of the people for whom they care. In this regard, charities and support groups also have an important role to play in offering safe places for caregivers to share experiences [34] and feel a renewed sense of control [29,30,31,32].

In terms of clinical practice, our findings suggest that UK lockdown restrictions had considerable negative effects on caregivers of PwP, meaning that a rapid restoration of services to pre-lockdown functioning is warranted—as also highlighted by Parkinson’s UK [37] and other patients and caregivers associations, all of which played a pivotal supporting role before and throughout the COVID-19 pandemic. This also appears to be in line with similar suggestions formulated around the world for caregivers of individuals with other chronic conditions such as mental health difficulties [38] and cancer [39], as well as other neurodegenerative diseases such as dementia [40], multiple sclerosis [41], and motor neurone disease [42]. Therefore, the urgent need to provide significant investments to support caregivers across multiple conditions, including Parkinson’s, is emphasised. Future studies should also aim to build on the present qualitative findings to inform more comprehensive mixed methods investigations of the impact of the pandemic on the lives of PwP’s caregivers.

## 5. Conclusions

The present study aimed to understand the subjective experiences of caregivers of PwP through and beyond the UK lockdowns during COVID-19. The findings show that loss of control and individual coping strategies were central to negative changes in the relationship dynamic between caregivers and their partners. In addition, the need to restore healthcare services and support is paramount not only for PwP and their caregivers but also for individuals affected by similar conditions. Ultimately, when considering the long-lasting negative effects of COVID-19 on healthcare access and availability, a simple restoration of pre-lockdown support levels does not appear sufficient to facilitate a successful return to optimal levels of support and wellbeing.

## Figures and Tables

**Table 1:** Participant and partner demographic information.

Pseudonym (Gender)	Age	Current (C)/ Former (F) Employment	Partner Age	Years since Partner’s Diagnosis	Length of Relationship (Years)
Paul (M)	75	Senior manager (F)	75	6	56
Gemma (F)	65	NHS worker (F)	64	12	---
Janine (F)	79	Healthcare worker (F)	82	15	50
Alice (F)	73	Personal assistant (F)	79	7	34
Sandra (F)	53	Civil servant (C)	56	9	32
Rachel (F)	64	Accountant (F)	71	11	48
Patrick (M)	57	Healthcare worker (C)	59	6	---
Jordon (M)	73	Senior practitioner (F)	74	20	57
Catherine (F)	57	Teacher (F)	67	9	21

Note. --- = information unavailable.

## Data Availability

The data presented in this study are available on request from the corresponding author. The data are not publicly available due to privacy restrictions.
